# Exploration of Metabolomic Markers Associated With Declining Kidney Function in People With Type 2 Diabetes Mellitus

**DOI:** 10.1210/jendso/bvad166

**Published:** 2023-12-22

**Authors:** Justina Krasauskaite, Bryan Conway, Christopher Weir, Zhe Huang, Jackie Price

**Affiliations:** Usher Institute, University of Edinburgh, EH8 9AG, Edinburgh, UK; Centre for Cardiovascular Science, The Queen's Medical Research Institute, Edinburgh BioQuarter, University of Edinburgh, EH16 4TJ, Edinburgh, UK; Usher Institute, University of Edinburgh, EH8 9AG, Edinburgh, UK; Usher Institute, University of Edinburgh, EH8 9AG, Edinburgh, UK; Usher Institute, University of Edinburgh, EH8 9AG, Edinburgh, UK

**Keywords:** chronic kidney disease, type 2 diabetes, metabolomics

## Abstract

**Background:**

Metabolomics, the study of small molecules in biological systems, can provide valuable insights into kidney dysfunction in people with type 2 diabetes mellitus (T2DM), but prospective studies are scarce. We investigated the association between metabolites and kidney function decline in people with T2DM.

**Methods:**

The Edinburgh Type 2 Diabetes Study, a population-based cohort of 1066 men and women aged 60 to 75 years with T2DM. We measured 149 serum metabolites at baseline and investigated individual associations with baseline estimated glomerular filtration rate (eGFR), incident chronic kidney disease [CKD; eGFR <60 mL/min/(1.73 m)^2^], and decliner status (5% eGFR decline per year).

**Results:**

At baseline, mean eGFR was 77.5 mL/min/(1.73 m)^2^ (n = 1058), and 216 individuals had evidence of CKD. Of those without CKD, 155 developed CKD over a median 7-year follow-up. Eighty-eight metabolites were significantly associated with baseline eGFR (β range −4.08 to 3.92; P_FDR_ < 0.001). Very low density lipoproteins, triglycerides, amino acids (AAs), glycoprotein acetyls, and fatty acids showed inverse associations, while cholesterol and phospholipids in high-density lipoproteins exhibited positive associations. AA isoleucine, apolipoprotein A1, and total cholines were not only associated with baseline kidney measures (P_FDR_ < 0.05) but also showed stable, nominally significant association with incident CKD and decline.

**Conclusion:**

Our study revealed widespread changes within the metabolomic profile of CKD, particularly in lipoproteins and their lipid compounds. We identified a smaller number of individual metabolites that are specifically associated with kidney function decline. Replication studies are needed to confirm the longitudinal findings and explore if metabolic signals at baseline can predict kidney decline.

The continuous growth in prevalence of type 2 diabetes mellitus (T2DM), currently reaching epidemic proportions [1], along with the demographic shift toward an aging population, means that the incidence of diabetic comorbidities is also rising rapidly [2, 3]. One of the most common of such health conditions is chronic kidney disease (CKD) [4], affecting more than a third of people with T2DM [5], and up to 37% of those are aged over 65 years [6]. Furthermore, CKD increases risk of progression to end-stage renal disease (ESRD) and mortality [7, 8] as up to 10% of deaths in this T2DM population are caused by renal failure [9]. Individuals diagnosed with T2DM and CKD face a 10-fold higher risk in 10-year cumulative all-cause death than those with T2DM alone [10] as well as increased risk of cardiovascular (CV)-related morbidities [11].

CKD is a long-term condition characterized by gradual yet irreversible loss of kidney function over time. Affected individuals require management of risk factors to slow the progression of kidney dysfunction and to prevent development of complications such as cardiovascular disease (CVD) [12, 13]. Initially CKD is clinically silent, which often delays diagnosis and prevents some opportunities for early intervention to slow its progression. Lack of treatment during the initial phase of disease development leads to accelerated deterioration to more advanced stages, which may also coincide with metabolic complications and CV-related outcomes. As a result, management of CKD and ESRD treatment are accompanied with high clinical burden and healthcare costs. For example, in 2009-2010, the cost of CKD management for the National Health Service in England was approximately £1.45 billion, of which 50% was used for treatment of ESRD, while remaining funds were spent on primary care costs, such as hypertension treatment [14].

Clinical screening programs offer routine measurement of kidney function for high-risk groups, including diabetes. Well-established biomarkers such as albuminuria or estimated glomerular filtration rate (eGFR) are routinely used; however, eGFR alone is often not specific enough as it is affected by factors unrelated to kidney dysfunction, such as age and muscle mass [15, 16], while a minority of older patients with CKD and T2DM have albuminuria [17]. Furthermore, these traditional biomarkers exhibit elevated levels only after substantial filtration capacity loss and/or kidney damage reaches more advanced levels [18]. Consequently, these markers rise in response to multiple injuries already sustained by renal cells and significant kidney function is lost [19, 20]. Recognizing CKD at an early stage is a crucial yet unmet medical requirement, which would enable targeted, early interventions. This is not only essential for predicting and preventing CKD onset but also for enhancing patient survival and minimizing associated morbidities, such as CVD mortality. Therefore, the quest for early biomarkers (ie, those that can predict the onset of CKD before significant changes in kidney function) remains imperative to fulfill this objective. This calls for further research that aims to discover biomarkers to improve prediction of disease onset, monitor disease progression, and increase understanding about the disease pathophysiology.

Analysis of the metabolome offers the prospect of finding novel prognostic biomarkers, and advancement of analytical tools such as nuclear magnetic resonance (NMR) spectroscopy provides an opportunity to investigate the metabolome in a high-throughput manner [21]. Many NMR studies have focused specifically on type 1 diabetes mellitus (T1DM) populations [22-26]. Three recent studies also used targeted NMR analysis to explore the associations of lipids, amino acids, and energy metabolites with kidney function in T2DM populations [27-29]. However, none of these studies explored the metabolite associations with incident CKD during the follow-up, defined according to the international guidelines Kidney Disease: Improving Global Outcomes (KDIGO) [4], which are widely used in clinical practice. Moreover, none of these studies focused exclusively on the older T2DM patients from a single cohort with longitudinal kidney function data.

Our study set out to explore the metabolomic profiles of kidney function in terms of associations between serum metabolites and kidney function decline using clinically relevant outcomes in a cohort of over 60-year-old T2DM subjects from the Lothian region of Scotland. Our objective was to discover new individual metabolites that merit additional investigation as potential contributors to biological disease pathways and/or for predictive assessment of CKD in individuals with T2DM.

## Materials and Methods

### Detailed Aims

Our first aim, in an explorative cross-sectional study, was to investigate the associations between individual metabolites and baseline kidney function [in terms of eGFR, urinary albumin to creatinine ratio (uACR), and CKD status] and to determine which metabolites were associated with kidney function independent of known CKD risk factors. To strengthen the evidence for a possible biological and/or predictive association, our second aim was to explore individual associations between the same metabolites and prospective outcomes (incident CKD and rapid decliner status); to identify associations, which were consistent with findings from the cross-sectional study; and to determine if altered metabolite levels might precede CKD onset.

### Participants and Baseline Clinical Assessments

We performed both cross-sectional and prospective analyses on the Edinburgh Type 2 Diabetes Study (ET2DS) [30]. Participants in the ET2DS were recruited between 2006 and 2007 from the Lothian diabetes register, a comprehensive database of people with T2DM living in the Lothian region of Scotland, whether managed in primary or secondary care. Men and women aged 60 to 74 with established T2DM were randomly selected by sex and 5-year age bands, and the resultant study population was shown to be largely representative of all people in this age group with T2D living in Lothian in terms of age, glycosylated hemoglobin (HbA1c), diabetes duration, and insulin treatment [31].

During the baseline clinic attendance, self-report questionnaires were collected, which included information about demographics and medical history. Fasting venous blood and urine samples were collected for biochemical analysis. Fresh samples were used to quantify serum creatinine, HbA1c, and uACR and were analyzed according to standard operating procedures in the Department of Biochemistry, Western General Hospital, Edinburgh, UK (Vitros Fusion chemistry system). The remainder of the samples were frozen for storage at −80 °C within 1 hour of venepuncture. The frozen serum samples were used to obtain metabolomics data using an automated high-throughput NMR platform at Nightingale Health Ltd (Kuopio, Finland) [32]. The panel consisted of 149 absolute metabolite concentrations.

Physical examinations were performed by trained research nurses. During physical examination, systolic and diastolic blood pressure (dBP) was measured in the right arm to the nearest 2 mmHg, with the participant in the supine position. To obtain the body mass index (BMI), height (in meters) was measured without shoes using a wall-mounted ruler and weight (in kilograms) was measured without outdoor clothing using electronic scales.

The ET2DS has been approved by the Lothian Medical Research Ethics Committee, and all participants provided written consent for clinical examinations and review of medical records.

### Outcomes

Kidney function was determined by eGFR, calculated from blood serum isotope dilution mass spetrometry–traceable creatinine using the Chronic Kidney Disease Epidemiology Collaboration equation [33]. Historical and follow-up serum creatinine measurements were obtained from the Lothian diabetes register, which included records from 2005 to May 2014. For cross-sectional analyses, continuous kidney function outcomes were eGFR and uACR, both measured at baseline. Reduced kidney function (referred to as baseline CKD) was defined as eGFR <60 mL min^−1^ (1.73 m)^−2^ in at least 2 measurements, 3 months apart. The results were reviewed at baseline and up to 2 years before baseline. In prospective analyses, new onset of reduced kidney function (referred to as incident CKD) was investigated in participants who had eGFR of at least 60 mL min^−1^ (1.73 m)^−2^ at baseline and up to 2 years before baseline. Incident CKD was defined as 2 eGFRs <60 mL min^−1^ (1.73 m)^−2^ at least 3 months apart during follow-up and at least 25% reduction in eGFR from baseline, as per the KDIGO guideline for the evaluation of CKD [19]. The annual rate of eGFR change was determined from linear eGFR slope that was estimated within each participant using a linear regression of eGFR against follow-up time in participants with a minimum of 1 year follow-up and 3 eGFR measurements. The estimates from linear regression slopes were converted into percentage annual rate of eGFR change with the following equation: percentage slope = (linear coefficient/baseline eGFR)*100. Rapid decliner status was defined as annual rate of eGFR change of at least −5%. The percentage decline was chosen over the absolute annual rate of decline as the former is less dependent on baseline eGFR.

### Statistical Analysis

Continuous variables were reported as means ± SD if normally distributed and as median with interquartile range if skewed (checked using histograms; data not included). Categorical variables were presented as total numbers with corresponding percentages. The 149 NMR measures were log-transformed and then standardized (subtraction of the mean and division by the SD). Values lower than the detection limit were assigned a value equivalent to half of the lowest observed value across the participants.

Cross-sectional study of associations between continuous baseline measures (eGFR and uACR levels) and individual metabolite concentrations was carried out using linear regression in all ET2DS participants. Baseline CKD status was analyzed using binary logistic regression. All of the regression models investigated the independent associations between individual metabolites and each outcome. The models were adjusted for potential confounders based on established CKD risk factors from a published risk prediction model [34] and included age, sex, diabetes control [diet only, tablets only, or insulin (with and without tablets/diet)], hypertension status (systolic blood pressure ≥140 and dBP ≥90 and/or use of blood pressure lowering medication), BMI, ever smoking status, and HbA1c. The metabolomic panel consisted of many lipoprotein and lipid measures, so the results may be affected by use of lipid-lowering medication. To test the robustness of the findings, we repeated the analysis for baseline eGFR with additional adjustment for medication use. Lastly, to explore if the observed associations could lie on the pathophysiological pathways of traditional CKD risk factors, the relationships between key metabolites and important covariates were assessed using Pearson correlations.

Similarly, in a prospective study that considered either incident CKD or rapid decliner status, each metabolite was modeled using logistic regression adjusting for baseline eGFR, age, and sex (Model 1) and Model 1 plus established CKD risk factors (Model 2).

To further explore the metabolomic profiles related to CKD phenotype and evaluate the stability of findings in traditional analyses, we employed a modern statistical technique—least absolute shrinkage and selection operator (LASSO). Since metabolomics data are characterized by high dimensionality and multicollinearity, LASSO was appropriate as it shrinks coefficients of some variables into exactly 0 by adding penalty, allowing selection of variables deemed most valuable for explaining variability in the outcome [35]. A 5-fold cross-validation incorporating all metabolites, sex, and age at baseline was applied to obtain the optimum tuning parameter lambda (λ) that gave the minimum mean residual error in the regression.

Regression estimates are reported with 95% confidence intervals. To account for large number of statistical tests, known to increase false-positive findings, the false discovery rate was controlled using the Benjamini-Hochberg method with up to 5% threshold for false-positive results [36]. The result was considered to be significant if multiple comparison adjusted P_FDR_ <0.05.

The analysis was done using R version 3.6.1 [37].

## Results

The baseline characteristics of ET2DS participants are summarized in Table 1. Those with baseline CKD were older and had higher BMI, longer diabetes duration, more hypertension, and prevalent CVD cases compared to those without baseline CKD. Also, those with baseline CKD had lower baseline eGFR, dBP, and total high-density lipoprotein (HDL) concentration. Among 1058 participants with available metabolomics data, 216 (20%) had CKD at baseline (see Supplementary Fig. S1 [38] for further details on kidney outcomes and Supplementary Table S1 [38] for details about sample size).

**Table 1.** Characteristics of ET2DS participants included study of metabolomic profiles subdivided according to their CKD status at baseline and characteristics of all ET2DS participants included

Clinical characteristic	CKD at baseline: no = 842	CKD at baseline: yes = 216	ET2DS BL 1058	*P*-value
Age (years)	67.48 (±4.13)	69.44 (±4.16)	67.88 (±4.21)	<.001
Sex: Male, n (%)	448 (53.2)	97 (44.9)	545 (51.5)	.029
Sex: Female, n (%)	394 (46.8)	119 (55.1)	513 (48.5)	
BMI (kg/m2)	31.08 (±5.64)	32.70 (±5.68)	31.41 (±5.69)	<.001
Diabetes duration (years)	7.455 (±5.98)	10.300 (±7.71)	8.034 (±6.47)	<.001
eGFR (ml min^−1^ (1.73 m)^−2^)	84.84 (±11.61)	48.70 (±12.92)	77.46 (±18.81)	<.001
uACR (mg/mmol)	1.12 [1.0.3]	1.49 [2.03]	1.16 [1.21]	<.001
HbA1c (%)	7.39 (±1.16)	7.464 (±1.01)	7.407 (±1.13)	.463
DBP (mmHg)	69.41 (±9.01)	67.619 (±8.86)	69.047 (±9.01)	.009
SBP (mmHg)	133.54 (±16.00)	131.91 (±17.84)	133.21 (±16.39)	.194
Total cholesterol (mmol/L)	4.32 (±0.93)	4.256 (±0.77)	4.31 (±0.90)	.340
HDL (mmol/L)	1.31 (±0.37)	1.21 (±0.32)	1.29 (±0.36)	<.001
Ratio-cholesterol:HDL	3.48 (±1.06)	3.71 (±1.13)	3.53 (±1.08)	.005
Hypertension, n (%)				<.001
Yes	665 (79.6)	204 (94.4)	869 (82.7)	
No	170 (20.4)	12 (5.6)	182 (17.3)	
History of CVD, n (%)				<.001
Yes	272 (32.3)	100 (46.3)	372 (35.2)	
No	570 (67.7)	116 (53.7)	686 (64.8)	
Smoker, n (%)				.032
Current	132 (15.7)	19 (8.8)	151 (14.3)	
Ex	391 (46.4)	105 (48.6)	496 (46.9)	
Never	319 (37.9)	92 (42.6)	411 (38.8)	
Diabetes control				<.001
Diet only n (%)	174 (21.3)	24 (11.3)	198 (19.3)	
Oral glucose lowering drugs only, n (%)	525 (64.4)	122 (57.3)	647 (62.9)	
Insulin use, n (%)	116 (14.2)	67 (31.5)	183 (17.8)	
Antihypertensive medication, n (%)				< .001
Yes	664 (79.5)	204 (94.4)	868 (82.6)	
No	171 (20.5)	12 (5.6)	183 (17.4)	
Lipid-modifying agents, n (%)				.018
Yes	709 (84.4)	196 (90.7)	905 (85.7)	
No	131 (15.6)	20 (9.3)	151 (14.3)	

Abbreviations: ACR, albumin to creatinine ratio; BL, baseline; BMI, body mass index; CKD, chronic kidney disease; CVD, cardiovascular disease; DBP, diastolic blood pressure; ET2DS, Edinburgh Type 2 Diabetes Study; eGFR, estimated glomerular filtration rate; HbA1c, glycated hemoglobin; HDL, high-density lipoprotein; HTN, hypertension; SBP, systolic blood pressure; uACR, urinary albumin to creatinine ratio.

Data presented are mean (±SD) or median [interquartile range]. The *P*-values for comparison of continuous variables for CKD and non-CKD groups was calculated using ANOVA (*t*-test with equal variances), and categorical variables were compared using chi-squared. Missing cases: Blood pressure medication n = 7; BMI n = 1; cholesterol n = 7; ratio cholesterol:HDL n = 8; diabetes duration n = 12; diabetes medications n = 30; HbA1c n = 9; HDL n = 7; hypertension status n = 7; lipid-lowering medication n = 2; sBP and dBP n = 2; uACR n = 12.

The description for 149 measured metabolites in the metabolomics panel and the mean values of each metabolite at baseline are shown in Supplementary Table S2 [38]. The average number of missing metabolite measurements was very low [0.03% (range 0-1.99%)].

### Cross-sectional Study

**Figure 1.**
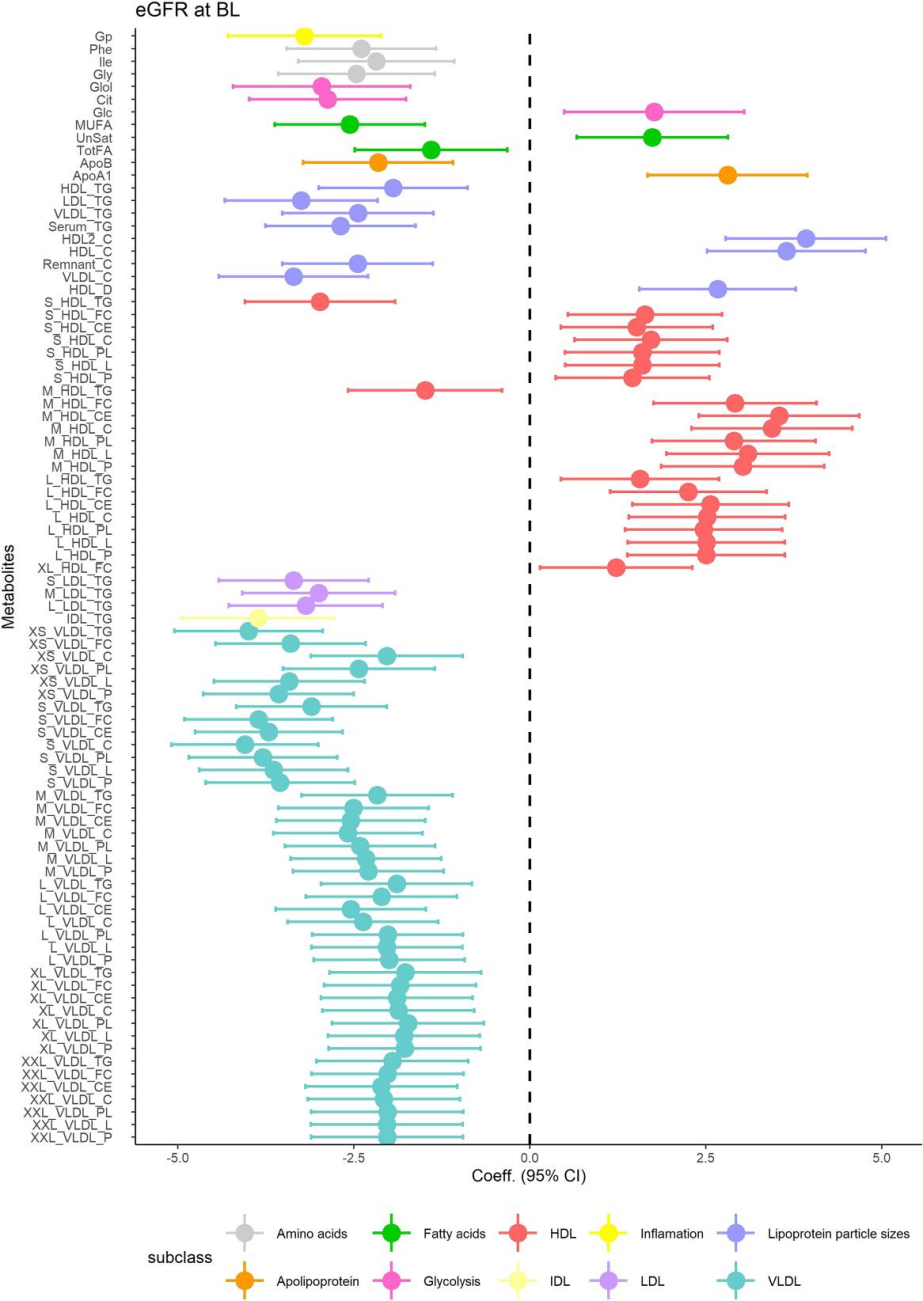
The cross-sectional associations between statistically significant metabolites (n = 88) and baseline measures of kidney function in terms of baseline eGFR (P _fdr_ < 0.05).

Abbreviations: CI, confidence interval; Coeff., β coefficients from linear regression; eGFR, estimated glomerular ratio; HDL, high-density lipoprotein; IDL, intermediate-density lipoprotein; LDL, low-density lipoprotein; OR, odds ratio; VLDL, very low density lipoprotein.

Linear regression analysis revealed 88 metabolites (excluding creatinine) significantly associated with baseline eGFR at P_FDR_ <0.05 (Fig. 1; see Supplementary Table S3 [38] for detailed *P*-values and βs). The wide confidence intervals suggested that the effect sizes lack precision due to limited sample size.

A majority of metabolites were inversely associated with eGFR. The most prominent association was with very-low-density lipoprotein (VLDL) subclasses, specifically cholesterol and triglyceride particles (β = −4.05, *P* = 4.8*10^−12^, β = −4.00 *P* = 9.9*10^−12^, respectively). Triglycerides in intermediate density lipoprotein and low-density lipoprotein, citrate, fatty acids, and inflammation-related glycoprotein acetyls as well as amino acids (AAs) phenylalanine, glycine, and isoleucine were also inversely associated with eGFR. Conversely, apolipoprotein A1 (ApoA1), total HDL cholesterol, and various lipid traits in large, medium, and small HDL subclass were positively associated with eGFR (P_FDR_ < 0.001). All of the significant associations (P_FDR_ < 0.05) remained the same in terms of significance and the effect direction after additional adjustment for lipid-lowering medication use, with an exception for free cholesterol in very large HDL, which only showed significant association with baseline eGFR before additional adjustment for lipid-lowering medication (Supplementary Table S4 [38]). Baseline CKD status was significantly associated with 88 metabolites (excluding creatinine), most of which reflected associations described for eGFR (Supplementary Table S5 [38], Supplementary Fig. S2 [38]). In total there were 80 metabolites associated with both outcomes, with the exception for 4 very large HDL particles (concentration of very large HDL, phospholipids, cholesterol, and cholesterol esters), which only showed significant association with CKD at baseline. On the other hand, free cholesterol in small HDL, glucose, total fatty acids, and estimated degree of unsaturation only showed significant association with baseline eGFR.

**Figure 2.**
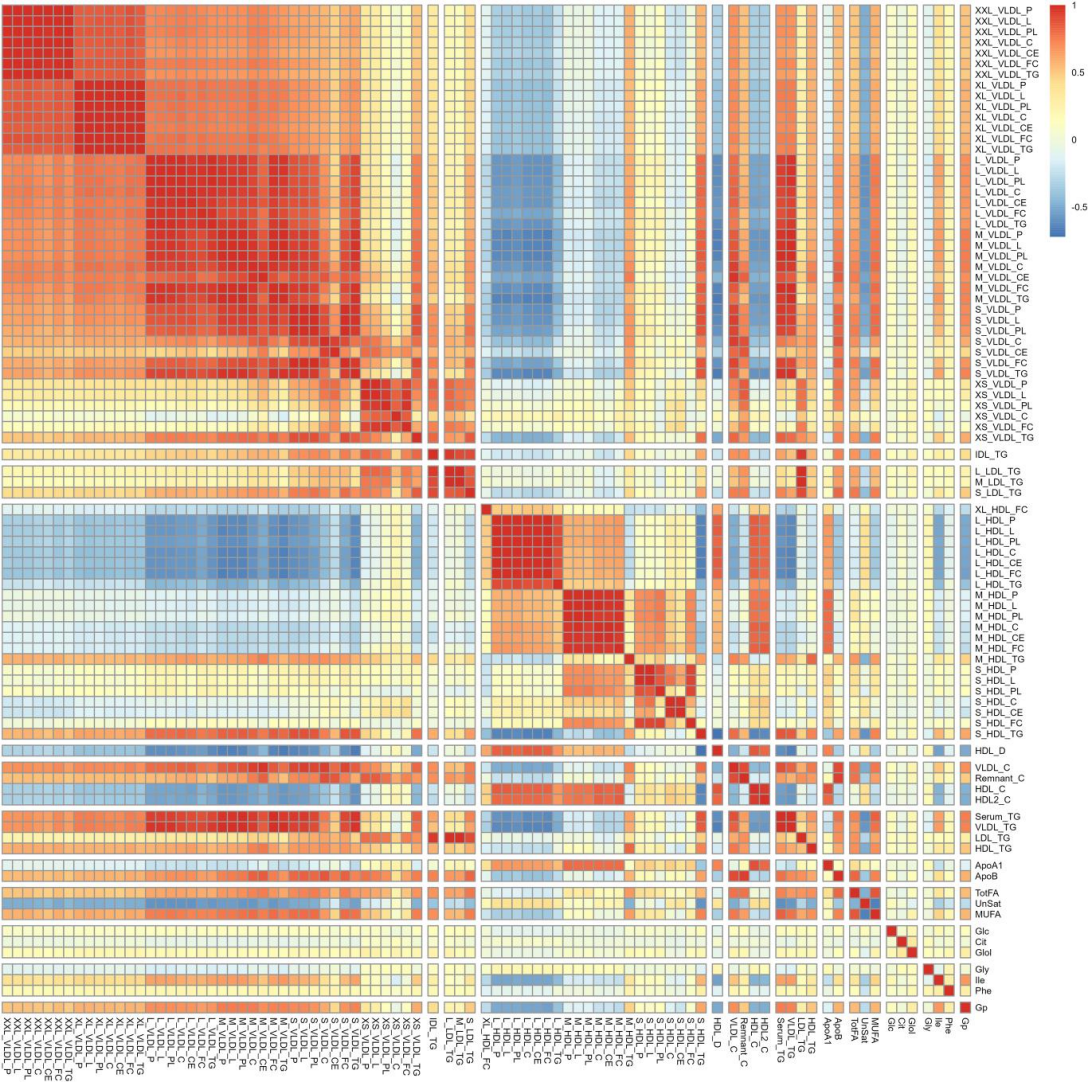
Correlation heat map between metabolites (standardized) that were significantly related to baseline eGFR in the Edinburgh Type 2 Diabetes Study (n = 88). Each small square on the X and Y axis depicts individual metabolites. Higher color intensity indicates high values of positive/negative pairwise correlation measure between metabolites.

Abbreviations: eGFR, estimated glomerular ratio.

Pearson correlation coefficients suggested high intracorrelation between significant metabolites (Fig. 2). Pearson correlation coefficients between important covariates and significant metabolites (Supplementary Fig. S3 [38]) showed weak-moderate correlations, with the expected exception for glucose-HbA1c since the latter is the marker of glucose control.

The uACR levels were significantly associated with only 2 metabolites (P_FDR_ < 0.05). Inflammation-related glycoprotein acetyls (mainly a1-acid glycoprotein) demonstrated a positive association with uACR (β=.10, P_FDR_ = 2.79*10^−2^). Glycoprotein acetyls were also associated with baseline eGFR and increased with worsening kidney function in terms of both uACR and eGFR. Aromatic AA tyrosine was negatively associated with uACR (β = −.11, P_FDR_ = 4.16*10^−3^) but was not significantly associated with eGFR (Supplementary Table S6 [38]).

In the complementary LASSO analysis, 9 metabolites were selected in association with baseline eGFR, 7 metabolites with baseline CKD status, and 5 metabolites for uACR on top of baseline age and sex (Supplementary Table S7 [38]). As expected, creatinine was selected in addition to a sparse panel of uncorrelated metabolites as part of the metabolomic profile of both baseline CKD, eGFR, and uACR (Supplementary Figs. S4, S5, S6 [38]). There was considerable overlap between metabolites selected in LASSO and the more traditional analysis (Suplementary Fig. S7 [38]). All metabolites that were significant in the linear regression analysis were also selected as part of the metabolomic profile in association to uACR. There was only 1 metabolite that was not significant in the traditional analysis that selected as part of the metabolomic profile of baseline CKD and the same for baseline eGFR profiles. Overall, this suggested that the associations detected in the regression analysis were robust.

### Prospective Study

Of 1047 participants with longitudinal eGFR data, 831 did not have evidence of CKD at baseline so were included in prospective study. Among them, 155 (19%) participants developed incident CKD during the median follow-up of 6.7 years [interquartile range 6.4, 7.1]. Of 1036 participants with sufficient follow-up eGFR data, the average rate of annual eGFR change was −1.2% (±4.4), and 157 (15%) were classed as rapid decliners.

**Figure 3.**
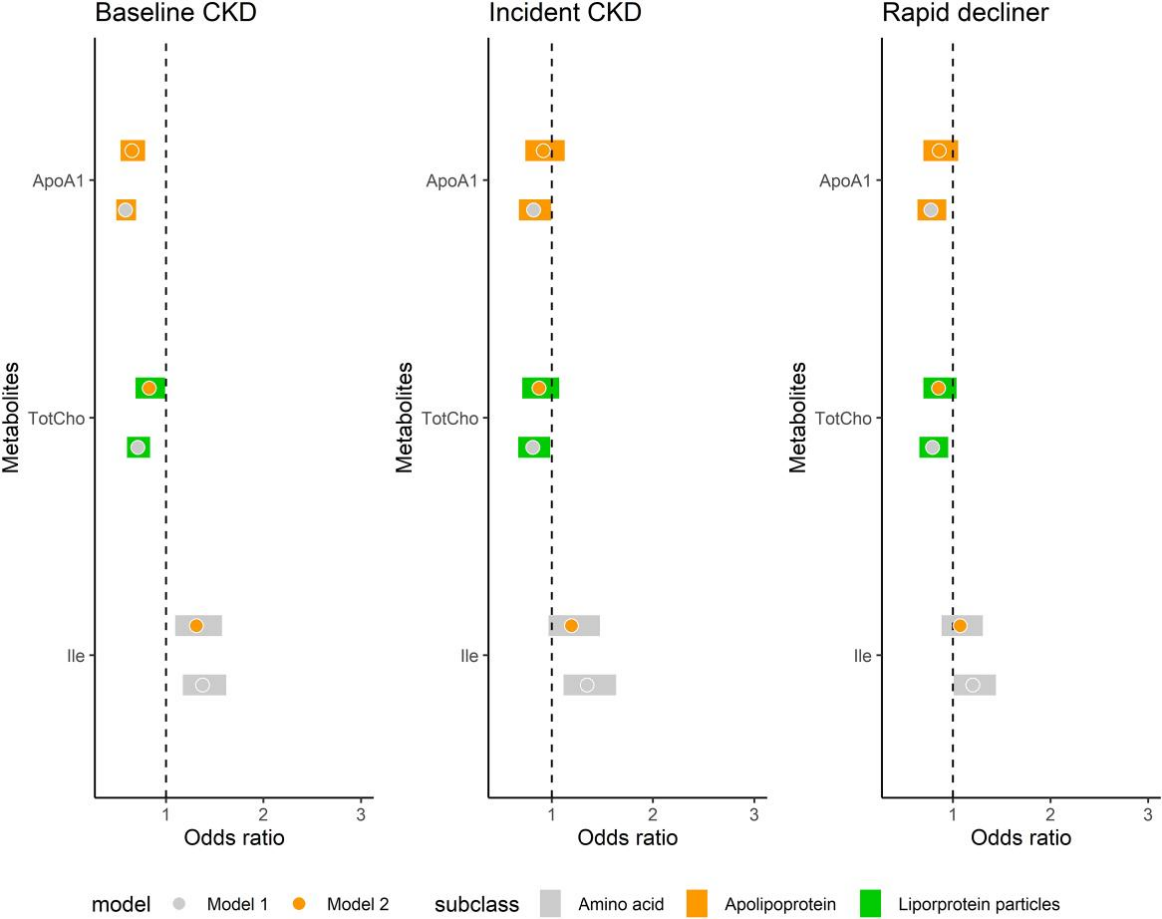
The key metabolites that showed stable associations with both cross-sectional and prospective outcomes. Left panel: associations with baseline CKD (P_FDR_ < 0.05, n = 1058); middle panel: associations with incident CKD (*P* < .05, n = 831, CKD events n = 155); right panel: associations with decliner status (*P* < .05, n = 1036). Model 1: adjusted for eGFR, age, and sex; Model 2: adjusted for Model 1+ CKD risk factors.

Abbreviations: ApoA1, apolipoprotein A1; CKD, chronic kidney disease; eGFR, estimated glomerular ratio; Ile, isoleucine; TotCho, total cholines.

The metabolites that showed some consistency in their association with both cross-sectional and prospective outcomes were AA isoleucine, total cholines, and ApoA1 (Fig. 3). These metabolites were significantly associated with baseline CKD (P_FDR_ < 0.05) even after adjustment for all CKD risk factors. While the associations with prospective outcomes were only at nominal significance (Model 1; see Supplementary Table S8 [38]) and were attenuated after adjustment for the full list of covariates (Model 2; see Supplementary Table S9 [38]), the direction and magnitude of associations remained stable.

After adjustment for an extensive list of CKD risk factors and multiple testing correction, none of the metabolites were significantly associated with CKD onset (P_FDR_ < 0.05). The metabolites that showed nominal association (*P* < .05) were not significantly associated with baseline eGFR or CKD, except for AA phenylalanine, where there was an apparent opposing direction of effect.

## Discussion

In this study, we described the associations of a large number of serum metabolites with kidney function-related outcomes in older Scottish individuals with T2DM. Baseline kidney function based on eGFR showed widespread associations with circulating metabolites, many beyond traditional panels. Among 88 significant findings (P_FDR_ < 0.05), VLDL and HDL subgroups, including various sizes and lipid compounds (eg, cholesterol, triglycerides, and phospholipids), exhibited the majority of the associations with baseline kidney function. Notably, 3 metabolites (isoleucine, ApoA1, and total cholines) were associated with both baseline CKD and longitudinal outcomes. However, none of the associations were statistically significant for prospective outcomes after correction for multiple testing and adjustment for known CKD risk factors, which suggested that none of the metabolites within this panel provide information on possible causal pathways related to CKD development. This aligns with results from previous studies that showed comparable associations with baseline eGFR (>100) but no significant associations with prospective outcomes [27-29]. Given the kidney's role as a central metabolite filtration unit, numerous baseline metabolite associations were anticipated, and indeed at least a few associations were found within each subgroup. The metabolites within subgroups are highly correlated, which ensured multiple particles from the same subgroup were linked to the outcome.

In contrast to baseline eGFR, only 2 metabolites, namely AA tyrosine and inflammation-related glycoprotein acetyls, were significantly associated with baseline uACR (P_FDR_ < 0.05). Considering uACR is a urinary biomarker, fewer correlations with circulating serum metabolites were expected than with eGFR (blood serum-based biomarker). Notably, previous studies that also considered metabolomic associations with uACR also reported fewer significant findings in people with either T2DM [27] or T1DM [39], possibly due to a smaller number of participants with available data on albuminuria. Nonetheless, we observed a potentially novel significant association between the glycoprotein acetyls and baseline uACR even after adjustment for CKD risk factors and multiple testing correction.

The metabolomic panel can be categorized into 3 distinct groups: amino acids, lipoproteins, and other particles.

### Amino Acids

The AAs isoleucine, phenylalanine, and glycine showed inverse association with baseline eGFR levels and increased the odds of having CKD at baseline. These AAs have been implicated in T2DM development [40-42]. Our analyses extend those of previous studies [27-29] in terms of effect direction, albeit with differences in magnitude, likely due to sample size variation [27-29].

Isoleucine, an essential branched chain amino acid (BCAA) [43], showed consistent associations in cross-sectional and prospective analyses, though was only nominally significant for incident outcomes and not independent of CKD risk factors. Higher isoleucine levels were linked to increased baseline CKD odds (P_FDR_ < 0.05), with the same direction for the incident outcomes (*P* < .05). In line with findings here, a small prospective study showed that higher isoleucine increased the odds of having rapid eGFR decline in T2DM subjects [44]. Furthermore, prior research found elevated isoleucine levels were associated with increased odds of having raised HbA1c (>53 mmol/L), indicating links with disrupted diabetes control [42]. Previous studies also showed that BCAAs, including isoleucine, were connected to diabetes [45] and insulin resistance [46, 47]. So it is possible that isoleucine could signal not only diabetes disruption and insulin resistance but also CKD development. Mechanisms involving increased gut microbiome-derived BCAAs may explain higher levels in insulin resistance [48]. Further exploration within T2DM may reveal pathophysiological insights and potential treatment targets.

Phe-to-tyrosine conversion happens not just in the liver but also in the kidney [49]. For example, circulating tyrosine was lower in patients with ESRD due to reduced conversion rates [49, 50]. Moreover, a number of explorative studies showed that tyrosine was associated with different eGFR categories in people with diabetes [51] and the general population [52] as well as CKD onset [53]. Although not significantly related to eGFR, tyrosine was inversely associated to baseline uACR (P_FDR_ < 0.05). This aligns with earlier findings associating tyrosine with diabetic nephropathy [28] and lower microvascular disease risk in T2D individuals [54]. This warrants further investigation of tyrosine as a prognostic marker of albuminuria.

### Lipoproteins

Patients with CKD often have increased cardiovascular risk as a result of dyslipidaemia, the hallmark of which is decreased HDL-cholesterol and increased triglyceride levels measured by traditional methods [55]. In agreement, we found NMR-measured total serum triglycerides and total cholesterol in HDL (HDL-C and HLD2-C) were associated with eGFR, suggesting typical traits of dyslipidaemia were linked with baseline CKD.

A more distinct dyslipidaemia pattern emerged via NMR profiling, where a number of nontraditional lipoproteins were associated with baseline kidney function, in some cases irrespective of lipid content. All sizes of VLDL particles with various lipid groups and triglycerides in multiple sizes of low-density lipoprotein showed inverse association, while various sizes of HDL with all its lipid compounds (except triglycerides) showed a positive association with baseline eGFR levels. These relationships were consistent with previous studies, which included comparative associations with many lipoproteins for both people with and without diabetes [27-29]. The results suggest that alterations of the lipoprotein composition and size beyond traditional markers of dyslipidaemia may contribute to the CV risk observed in CKD patients.

Consistent with previous studies [27, 28], associations remained even after accounting for lipid-lowering medication use. Notably, statin therapies do not fully address increased triglycerides and decreased HDL levels [56]. The variations in lipoprotein composition observed in CKD patients may impact CVD risk and influence lipid-lowering treatment effectiveness.

### Other Metabolites

Inflammation-related glycoprotein acetyls was 1 of 2 metabolites significantly associated with uACR at baseline after adjustment for all CKD risk factors. Tofte et al observed associations between glycoprotein acetyls and uACR; however, this association did not persist after correcting for multiple testing. It is worth noting that the aforementioned study had a notably smaller number of participants with uACR data, suggesting the possibility of insufficient cases to detect significant findings. Interestingly, earlier studies have demonstrated that glycoprotein acetyls are associated with an increased risk of T2DM, cardiovascular disease, renal failure, and other conditions related to large internal organs [57]. These associations, however, have not been demonstrated within the T2DM population before. Therefore, our study reveals potentially novel associations between albuminuria and glycoprotein acetyls in the T2DM population, warranting further investigation to confirm whether these associations also contribute to the risk of developing albuminuria. ApoA1 is a primary apolipoprotein contained in the HDL particle, and both of these are known to be associated with reduced CVD risk. HDL composition and function in people with CKD is altered, leading to reduced levels of ApoA1 [58]. In agreement with previous studies, we also found ApoA1 was positively associated with baseline eGFR [27-29]. Albeit only at a nominal significance level, ApoA1 also showed consistent inverse association with baseline CKD, incident CKD, and decliner status, suggesting that higher levels of ApoA1 were associated with reduced odds of CKD.

Lastly, higher levels of total cholines also significantly reduced odds of baseline CKD independent of CKD risk factors (P_FDR_ < 0.05) and demonstrated consistent direction of effect in terms of incident CKD and decliner status. In contrast, previous studies showed an opposite relationship, where higher levels of cholines were associated with reduced eGFR in T2DM and general populations [29]. Moreover, cholines demonstrated strong inverse association with GFR [59] and predicted CKD onset at 8-year follow-up [60]. Cholines are acquired from dietary sources [61], so total choline levels are directly influenced by consumption of meat, fish, and eggs. Generally, older populations tend consume fewer protein-rich foods; thus, conflicting results may be due to fundamental demographic differences in the populations studied. This may suggest differences between age groups in metabolic profiles and the importance of considering implications of age and diet when studying the metabolome.

### Strengths and Limitations

To our best knowledge, the ET2DS is the largest single cohort study that explores associations between incident CKD and this NMR metabolomic panel specifically in an older T2DM population. Although 3 studies analyzed a similar NMR panel [27-29], they did not focus on this particular age group, who may be most at risk of developing CKD. Furthermore, the studies of Tofte et al [27] and Barrios et al [28] were a meta-analysis of smaller cohorts, likely chosen based on NMR panel availability. This is not ideal as results may be affected by heterogeneity between cohorts that was not accounted for (eg, systemic differences involved in participant recruitment, preanalytical influences on metabolomic measurements).

A key strength of our study is the availability of medical records, which included kidney function measurements from 2 years before baseline to 7 years of follow-up. These data enabled us to apply a CKD definition in line with KDIGO to determine clinically relevant CKD cases. This is preferable to dichotomizing the single-point kidney function measurements to define CKD phenotype, which may lead to misclassification-caused temporal variability of eGFR [62]. Another important advantage of the present study is a well-characterized and representative ET2DS cohort. The wealth of demographic and clinical variables available enabled analysis with adjustment for an extensive list of covariates and allowed identification of associations independent of established risk factors.

We also note some study limitations. First, there was no comparison to general or T1DM populations; thus we were unable to ascertain which metabolic traits were associated with CKD specifically in T2DM [31]. Second, the statistical power of the analyses was not optimal considering the large number of exposures tested and the relatively low number of events, compromising the utility of multiple testing correction (ie, in an underpowered study, multiple testing correction may eliminate important true findings). In light of this, results from the prospective analysis with nominal significance levels (*P* < .05) were considered to generate hypotheses for further study. Third, there was no comparable external population to replicate our findings, which is particularly important for machine learning methods like LASSO, which tends to over fit the data and provide optimistic results. A future replication study is therefore warranted to evaluate if the findings are generalizable to other populations of older people with T2DM. Fourth, the cross-sectional analysis was based on a single measure of kidney function. Considering that both eGFR and uACR may be subject to daily variability, the single measures may not be reliable representations of the actual kidney function. However, the more reliable measure, less prone to daily variation, is the measured GFR (which is determined through direct measurement of kidney clearance, typically using substances like inulin, iothalamate, or radioisotopes). However, measured GFR is more invasive and generally not widely used in clinical or research practice. Nonetheless, we did take into consideration the variability of a single measure in eGFR and used a more thorough definition of incident CKD in the prospective analysis.

In conclusion, the present cohort study of older T2D subjects revealed widespread changes within the metabolomic profile of CKD, particularly in lipoproteins and their lipid compounds. The traits in the HDL subclass were increased in those with higher kidney function, while VLDL, triglycerides, and some AAs were increased in those with lower kidney function. Replication studies are needed to confirm the longitudinal findings and clarify if the metabolic signals at baseline predict future kidney decline.

## Data Availability

Some or all datasets generated during and/or analysed during the current study are not publicly available but are available from the corresponding author on reasonable request.
